# Proteomic Characterization of Corneal Epithelial and Stromal Cell-Derived Extracellular Vesicles

**DOI:** 10.3390/ijms251910338

**Published:** 2024-09-26

**Authors:** Vincent Yeung, Nikolay Boychev, Levi N. Kanu, Veronica Ng, Amy E. Ross, Audrey E. K. Hutcheon, Joseph B. Ciolino

**Affiliations:** Department of Ophthalmology, Schepens Eye Research Institute of Mass Eye and Ear, Harvard Medical School, Boston, MA 02114, USA; nboychev@meei.harvard.edu (N.B.); levi_kanu@meei.harvard.edu (L.N.K.); ngve@bc.edu (V.N.); amy_ross@meei.harvard.edu (A.E.R.); audrey_hutcheon@meei.harvard.edu (A.E.K.H.); joseph_ciolino@meei.harvard.edu (J.B.C.)

**Keywords:** cornea, extracellular vesicles (EVs), epithelial cells, fibroblast, ingenuity pathway analysis, keratocytes, myofibroblast, proteomics, wound healing

## Abstract

Communication between the different layers of the cornea (epithelium and stroma) is a complex, yet crucial element in the corneal healing process. Upon corneal injury, it has been reported that the bi-directional cross talk between the epithelium and stroma via the vesicular secretome, namely, extracellular vesicles (EVs), can lead to accelerated wound closure upon injury. However, the distinct protein markers of EVs derived from human corneal epithelial (HCE) cells, keratocytes (HCKs), fibroblasts (HCFs), and myofibroblasts (HCMs) remain poorly understood. All EVs were enriched for CD81 and showed increased expression levels of ITGAV and FN1 in HCM-EVs compared to HCE- and HCF-EVs. All EVs were negative for GM130 and showed minimal differences in biophysical properties (particle concentration, median particle size, and zeta potential). At the proteomic level, we show that HCM-EVs are enriched with proteins associated with fibrosis pathways, such as COL6A1, COL6A2, MMP1, MMP2, TIMP1, and TIMP2, compared to HCE-, HCK-, and HCF-EVs. Interestingly, HCE-EVs express proteins involved with the EIF-2 signaling pathway (stress-induced signals to regulate mRNA translation), such as RPS21, RALB, EIF3H, RALA, and others, compared to HCK-, HCF-, and HCM-EVs. In this study, we isolated EVs from cell-conditioned media from HCE, HCKs, HCFs, and HCMs and characterized their biophysical and protein composition by Western blot, nanoparticle tracking analysis, and proteomics. This study supports the view that EVs from the corneal epithelium and stroma have a distinct molecular composition and may provide novel protein markers to distinguish the difference between HCE-, HCK-, HCF-, and HCM-EVs.

## 1. Introduction

The cornea is the most anterior tissue of the eye that transmits light and provides protection to the intraocular eye components [1]. The outermost layer is the (human) corneal epithelium (HCE), composed of non-keratinized squamous epithelial cells. Underneath the corneal epithelium is the corneal stroma, which consists of a highly organized extracellular matrix (ECM) that plays a vital role in cell migration, differentiation, and maintenance of the epithelium [2]. The (human) corneal keratocytes (HCKs) are a population of neural crest-derived, quiescent cells of the stroma that contribute to the ECM and are responsible for secretion of collagen fibrils and proteoglycans (PGs) [3]. HCKs have the capacity to transform into a repair phenotype of activated human corneal fibroblasts (HCFs) following stromal injury [4]. HCFs are a proliferative and migratory cell type that produces repair-type ECM components that include fibronectin (FN1), integrins, and proteinases [5]. Upon appropriate inducible conditions in the presence of serum and transforming growth factor β1 (TGF-β1), HCFs can undergo further differentiation into human corneal myofibroblasts (HCMs) [6]. HCMs can be characterized by the presence of alpha smooth muscle actin (αSMA) and increased contractile fibers and cell size [7]. The HCMs are responsible for secreting a variety of soluble factors, such as matrix metalloproteinases (MMPs), tissue inhibitor of metalloproteinase (TIMP), collagen, and others, that contribute to ECM remodeling and collectively mediate wound closure [5], although the persistent activation of HCMs can lead to corneal scarring, diminished corneal transparency, and vision loss.

In the cornea, cross talk between the corneal epithelium and stroma plays a vital role in regulating corneal wound repair and infection states, and it is understood that cell–cell communication is critical for this process [8,9,10,11,12]. This cross talk is mediated by paracrine factors such as soluble cytokines, but specifically extracellular vesicles (EVs) have become prominent relative to physiological and pathological responses to wound healing [8,13]. Initial studies from our laboratory provided early evidence that following a keratectomy, EVs are released into the corneal stroma. Additional studies have reported that corneal epithelium-derived EVs are involved in corneal wound healing by promoting neovascularization [14]. Recently, we have shown that HCE-EVs can trigger HCFs into αSMA+ HCM [5], highlighting that these studies report different roles for how HCE-EVs affect the corneal stroma.

To date, our current understanding of EVs has been documented to express proteins such as tetraspanins and others. With a focus on the cornea, there lack studies documenting the specific protein markers that the corneal epithelium and stroma-derived EVs express. In this study, we isolated and characterized EVs from the HCE, HCKs, HCFs, and HCMs to understand the differences in their biophysical and molecular composition. We performed proteomic analysis and ingenuity pathway analysis (IPA) to understand the differences in their EV cargo and whether it contributes to distinct diseases and biological pathways. Our study shows that EVs derived from the HCE, HCKs, HCFs, and HCMs contain a unique array of proteins distinct to each cell type and may serve to act as novel protein markers for future studies. Understanding the differences in EV cargo will strengthen our knowledge gap with regard to the molecular profile of HCE-, HCK-, HCF-, and HCM-EVs in corneal wound healing.

## 2. Results

### 2.1. Characterization of HCE-, HCK-, HCF- and HCM-EVs

**Figure 1:**
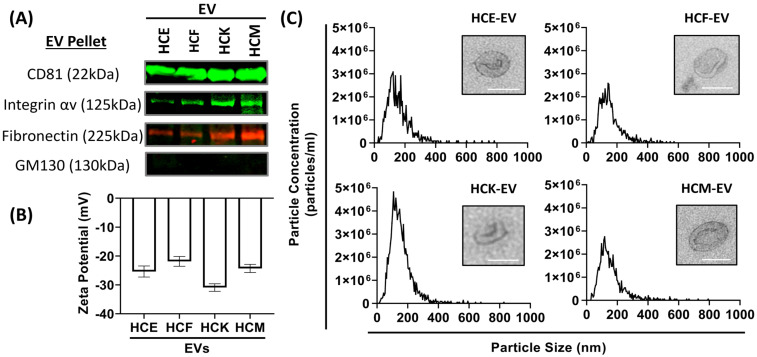
Characterization of human corneal epithelium (HCE)-, fibroblast (HCF)-, keratocyte (HCK)-, and myofibroblast (HCM)-derived extracellular vesicles (EVs). (**A**) 20 μg of EV pellets were loaded into each lane and analyzed by Western blot to probe for CD81, integrin αv, fibronectin, and a negative control (GM130). (**B**) Zeta (ζ) potential of EV pellets was measured in EV samples using NTA with Zetaview™. (**C**) Size distribution histogram for each EV sample was constructed using nanoparticle tracking analysis (NTA) with representative average values shown. (**C inset**) Transmission electron microscopy images demonstrating EV morphology (high magnification, 49,000×, scale bar: 100 nm). n = 3 independent EV preparations.

To investigate whether EVs were isolated from the HCE, HCKs, HCFs, and HCMs, we used an EV isolation protocol [2] and followed the guidance from MISEV2023 to determine the minimal data required for studying EVs (MISEV2023) [15]. Isolated EVs were normalized to protein concentration for this study. We determined protein expression by Western blotting by probing for CD81, integrin αv, fibronectin, and GM130. We highlight that CD81 expression levels were similar across the HCE-, HCK-, HCF-, and HCM-EV samples. Interestingly, we show elevated integrin αv expression in ascending order from HCE-, HCK-, and HCF- to HCM-EVs (Figure 1A). We also probed for fibronectin to evaluate whether an expression profile akin to integrin αv was more abundant in HCM-EVs compared to others. All samples showed negative expression of GM130, indicative of no contaminants. We characterized the biophysical properties of EVs by performing Zetaview and NTA measurements to show there were no differences between any EV types, with a net negative surface charge ranging from −25.87 mV to −39.46 mV indicating colloidal EV stability (Figure 1B), with all nanoparticle measurements ranging between 100 and 150 nm in size (Figure 1C), and EV morphology by TEM. The data suggested that there are no differences in their biophysical properties, but also showed subtle differences in protein expression.

### 2.2. Proteomic Characterization of HCE-EVs

**Figure 2:**
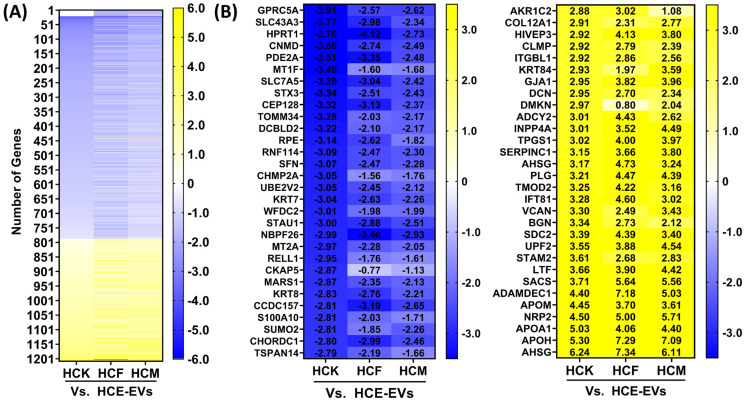
Proteomic analysis of HCE-EVs. (**A**) Heatmap of expressed proteins from HCE-EVs relative to HCF-, HCK-, and HCM-EVs. A multi-group comparison was performed and showed that 1209 proteins were differentially expressed in our dataset. Proteins selected show log2 fold-change values (blue (−3.5) to yellow (+3.5) through white), ranked by adjusted *p* < 0.05. (**B**) The top 30 proteins selected from this heatmap with decreased (blue (−3.5)) and increased (yellow (+3.5)) log2 fold changes in HCF-, HCK-, and HCM-EVs relative to HCE-EVs. Data shown as n = 3 independent EV preparations.

To compare the proteomic protein repertoire of HCE-, HCK-, HCF- and HCM-EV samples (pooled from three independent preparations), samples were digested in solution and TMT-labeled peptides were analyzed by nanoLC-MS/MS on a high-resolution mass spectrometer. Overall, 2482 peptides were identified from all EV samples, and further details are specified in Supplemental Table S1. Comparisons in peptides with log2 fold changes (<−0.5 or >0.5) for HCE- vs. HCK-, HCF-, and HCM-EVs were confirmed. On multi-group comparison, 1209 proteins were found to have significant log2 fold changes based on a false-discovery rate (FDR) adjusted *p*-value of <0.05 (Figure 2A). Whilst the magnitude of change was dissimilar for many proteins, the heatmap (Figure 2B) shortlists the top 30 expressed proteins with >0.5 log2 fold increase in HCE-EVs compared to HCF-, HCF-, and HCM-EVs, such as GPRC5A, KRT7, KRT8, WFDC2, and others. Other enriched HCE-EV proteins also included KRT18 and KRT19 (data in Supplemental Table S1). We extended our observations to highlight the top 30 proteins with <−0.5 log2 fold decrease in HCE-EVs compared to other EV samples, such as COL12A1, ADCY2, SDC2, TMOD2, and others. To gain functional insight into the proteomic cargo of HCE-EVs, we performed IPA for the top disease and biological functions enriched in HCM-EVs. This highlighted that the HCE-EV-expressed proteins were associated with the EIF2 signaling pathway, which is known to initiate stress-related signals to regulate mRNA translation (an array of RPS- and EIF-related proteins) [16] (Supplemental Figure S1). This indicates that HCE-EVs have a distinct functional feature compared to HCF-, HCK-, and HCM-EVs.

### 2.3. Proteomic Characterization of HCK-EVs

**Figure 3:**
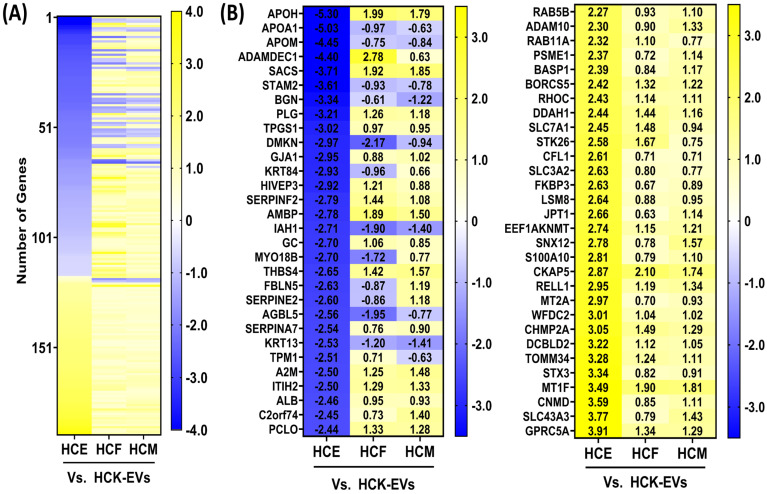
Proteomic analysis of HCK-EVs. (**A**) Heatmap of expressed proteins from HCK-EVs relative to HCE-, HCF-, and HCM-EVs. A multi-group comparison was performed and showed that 1209 proteins were differentially expressed in our dataset. Proteins selected show log2 fold-change values (blue [−3.5] to yellow [+3.5] through white), ranked by adjusted *p* < 0.05. (**B**) The top 30 proteins selected from the heatmap with decreased (blue [−3.5]) and increased (yellow [+3.5]) log2 fold changes in HCE-, HCF-, and HCM-EVs relative to HCK-EVs. Data shown as n = 3 independent EV preparations.

To understand the unique proteomic protein repertoire of HCK-EVs compared to HCE-, HCF-, and HCM-EV samples (pooled from three independent preparations), a multi-group comparison was conducted. We found that 190 proteins had significant log2 fold changes based on an FDR-adjusted *p*-value of <0.05 (Figure 3A). In Figure 3, we highlight the top 30 expressed proteins with >0.5 log2 fold increase in HCK-EVs compared to HCE-, HCF-, and HCM-EVs, such as APOA1, APOM, DMKN, IAH1, AGBL5, and KRT13, as well as the top 30 proteins with <−0.5 log2 fold decrease in HCK-EVs compared to other EV types, including RAB5A, RAB11A, CHMP2A, ADAM10, and others.

### 2.4. Proteomic Characterization of HCF-EVs

**Figure 4:**
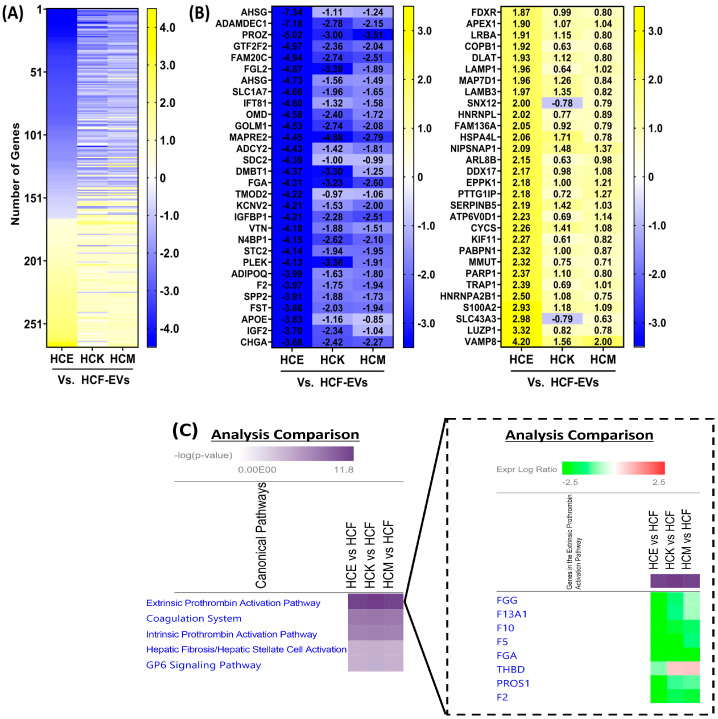
Proteomic analysis of HCF-EVs. (**A**) Heatmap of expressed proteins from HCF-EVs relative to HCE-, HCK-, and HCM-EVs. A multi-group comparison was performed and showed that 269 proteins were differentially expressed in our dataset. Proteins selected show log2 fold-change values (blue (−3.5) to yellow (+3.5) through white), ranked by adjusted *p* < 0.05. (**B**) The top 30 proteins selected from the heatmap with decreased (blue (−3.5)) and increased (yellow (+3.5)) log2 fold changes in HCE-, HCK- and HCM-EVs relative to HCF-EVs. Data shown as n = 3 independent EV preparations. (**C**) The top disease and biological functions identified from IPA using the dataset of 269 protein with the extrinsic prothrombin activation pathway highlighted. The top-scoring proteins were identified in HCF-EVs, green indicating elevated expression and red indicating lower expression compared to other EVs. Fisher’s exact test was used for determination of enrichment, and *p*-values less than 0.05 were taken as significant.

We explored the expression of proteins unique to HCF-EVs compared to HCE-, HCK-, and HCM-EV and found that 269 proteins had significant log2 fold changes based on an FDR-adjusted *p*-value of <0.05 on a multi-group comparison (Figure 4A). The expression of many proteins was dissimilar; however, we show the top 30 expressed proteins with >0.5 log2 fold increase in HCF-EVs compared to HCE-, HCK-, and HCM-EV, such as PROZ, SDC2, TMOD2, VTN, APOE, and others, in Figure 4B, as well as the top 30 proteins with <−0.5 log2-fold decrease in HCF-EVs compared to HCF-, HCF-, and HCM-EVs, such as FDXR, APEX1, LAMP1, PARP1, VAMP8, and others. To gain functional insight into the proteomic cargo of HCF-EVs, IPA revealed that the extrinsic prothrombin activation pathway, which is involved in the corneal wound healing process [17], was involved, which indicates that a functional pathway is associated with HCF-EVs (Figure 4C).

### 2.5. Proteomic Characterization of HCM-EVs

**Figure 5:**
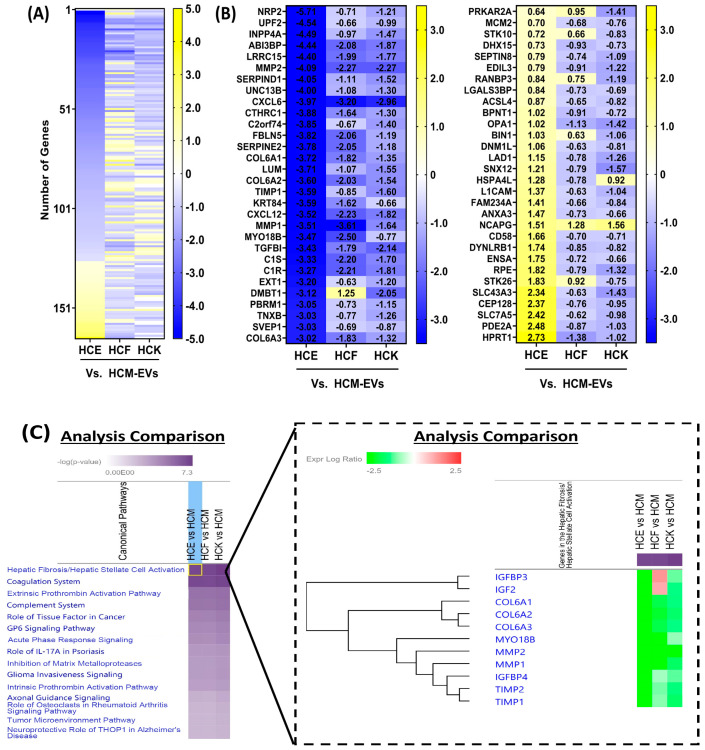
Proteomic analysis of HCM-EVs. (**A**) Heatmap of expressed proteins from HCM-EVs relative to HCE-, HCF-, and HCK-EVs. A multi-group comparison was performed and showed that 269 proteins were differentially expressed in our dataset. Proteins selected show log2 fold-change values (blue (−3.5) to yellow (+3.5) through white), ranked by adjusted *p* < 0.05. (**B**) The top 30 proteins selected the heatmap with decreased (blue (−3.5)) and increased (yellow (+3.5)) log2 fold changes in HCE-, HCF-, and HCK-EVs relative to HCM-EVs. Data shown as n = 3 independent EV preparations. (**C**) The top disease and biological functions were identified from IPA using the dataset of 269 proteins with the extrinsic prothrombin activation pathway highlighted. The top-scoring proteins were identified in HCM-EVs, green indicating elevated expression and red indicating lower expression compared to other EVs. Fisher’s exact test was used for determination of enrichment, and *p*-values less than 0.05 were taken as significant.

We explored the profile of proteins distinct to HCM-EVs relative to HCE-, HCF-, and HCK-EVs. We found that 166 proteins had significant log2 fold changes based on an FDR-adjusted *p*-value of <0.05 by a multi-group comparison (Figure 5A). The top 30 proteins with >0.5 log2 fold increased expression in HCM-EVs compared to HCE-, HCF-, and HCK-EV included COL6A1, COL6A2, COL6A3, MMP1, MMP2, CXCL6, CXCL12, and others (Figure 5B). Among the top 30 proteins with <−0.5 log2 fold decrease in HCM-EVs compared to HCE-, HCF-, and HCK-EVs was NCAPG. Interestingly, the distinct expression profiles highlighted instances where there was <−0.5 log2 fold decrease in HCM-EVs compared to HCE- and HCF-EVs, such as PRKAR2A, STK10, RANBP3, BIN1, and STK26, but this was not the case when compared to HCK-EVs. IPA revealed that the hepatic fibrosis pathway, involved in increased expression and deposition of the ECM [18], was activated in HCM-EVs (Figure 5C).

## 3. Discussion

The human corneal epithelium has been extensively characterized, with research stemming from the human immortalized Araki-Sasaki cells [19] and further studies that discussed other protein markers of the corneal epithelium, such as cornea-specific keratin and adhesion proteins and stem cell and differentiation proteins [20]. The corneal stroma has been studied to understand how quiescent HCKs maintain corneal transparency and HCFs and subsequent HCMs protect the corneal stroma from various external stimuli such as mechanical injury or pathogens [21]. HCMs are defined by the expression of αSMA+ fibers and other related ECM proteins (MMPs, TIMPs, collagen and others) that govern their wound closure/repair phenotype [18]. However, there remains a knowledge gap in understanding how soluble secretomes, namely, EVs, from these cell types differ in their expression profile and their cells of origin.

In our present study, EV normalization strategies differed between cell equivalence, particle number, and lipid concentration, but we normalized all experiments to protein concentration. As reported in our previous studies [2,22], protein normalization eliminates any bias towards a different metric, and this should reflect a fair comparison in protein expression in the EV phenotype. Here, we isolated HCE-, HCK-, HCF-, and HCM-EVs and characterized their biophysical properties and their proteomic protein properties. We found minimal differences in their biophysical properties when assessing size distribution profile measurements, particle concentration, median size of particles, zeta potential, and EV morphology. At the protein level, we found that expression levels of CD81 were consistent across all EV sample types, but interestingly showed elevated integrin αv expression in ascending order from HCE-, HCK-, and HCF- to HCM-EV. Fibronectin expression patterns in EVs were akin to integrin αv, with an abundance in HCM-EVs compared to others. All samples showed negative expression of GM130, indicative of no contaminants. These data suggest that EVs were isolated and characterized in a way that was aligned with the minimal information for studies of EVs (MISEV2023) guidelines [15].

Our proteomic data showed the expression profile of HCE-, HCK-, HCF-, and HCM-EVs had overlapping proteins, but enriched proteins were found in distinct subtypes. With regard to the HCE, the keratin (KRT) proteins are a vital intermediate filament family that helps form a complete network of cytoskeletal structure. This has been investigated, and different corneal epithelial studies have documented that it expresses KRT [23,24,25]. Our data suggest that KRT18 and KRT19 were enriched in HCE-EVs, and similar studies have reported the presence of KRT18/KRT19 expression in the corneal epithelium [26,27]. HCE-EVs were also enriched in KRT7 and KRT8, which were also found on epithelial cells in a corneal epithelial regeneration mouse model study [28].

We found that WFDC2 and GPRC5A were enriched in HCE-EVs, which is a new observation. The functional role of WFDC2 and GPRC5A has not been reported in the corneal epithelium. GPRC5A is enriched in HCE-EVs compared to corneal stromal EVs and is an orphan G protein-coupled receptor (GPCR) that is part of signaling pathway that modulates adhesion signaling via FAK/Src/Rho GTPases [29]. This study highlights GPRC5A’s role in the regulation of ITGB1-mediated epithelial cell adhesion, and similar studies also support the notion of GPRC5A’s role in epithelial cell migration and proliferation, with similar findings being reported in different disease models [30,31]. The WFDC2 protein is a highly expressed family member in differentiated epithelial cells, highlighting its presence in secretory cells of the control airway epithelium and also in submucosal glands [32]. Here, we report that WFDC2 and GPRC5A expression may be a novel marker for the corneal epithelial cells and subsequent EVs, but their functional role has yet to be determined, with WFDC2 and GPRC5A reported only in non-corneal disease models.

With an emphasis on HCM-EVs, we highlighted 166 proteins as being significantly different compared to HCE-, HCK-, and HCF-EVs. We found elevated expression in HCM-EV proteins such as COL6A1, COL6A2, COL6A3, MMP1, MMP2, TIMP1, TIMP2, CXCL6, CXCL12, and others from our proteomic analysis. Collectively, from our IPA, these proteins are associated with the activated fibrosis pathway (or hepatic stellate pathway by IPA). Collagen type VI is a non-fibrillar heterotrimeric protein expressed in the ECM that is associated with end-to-end creation of collagen VI microfibrils organized in a microfilamentous network, and is encoded by COL6A1, COL6A2, and COL6A3 [33,34]. In patient samples with fibrotic models, such as arthrofibrosis, liver fibrosis, or idiopathic fibrosis, it has been commonly reported that collagen VI colocalizes with an αSMA-positive myofibroblast localized to the fibrotic foci [35,36,37,38]. This aligns with observations of elevated MMP1 and MMP2 driving remodeling of the ECM and also CXCL6 and CXCL12 driving fibroblast collagen synthesis [39,40,41]. These are characteristics of the myofibroblastic secretomes that are associated with the activated fibrosis pathway [42,43]. These data reveal that HCM-secreted EVs that retain some of their protein markers of cell origin are likely to invade and repair injured tissue by secreting and reorganizing the ECM, yet persistent secretion of HCM-EVs may lead to excessive and visually disabling scarring that interlinks with fibrotic signaling pathways.

There were some limitations within this study. The proteins associated with HCM-EVs could be vital for triggering fibrotic pathways, although this can only be deemed as speculative, as it would require further investigation. Therefore, to provide greater context, in future studies, we will employ assays to demonstrate HCM-EV-associated proteins’ functional activity. Furthermore, we cannot dismiss the unique cargo that EVs might encapsulate, such as lipids and different forms of nucleic acids, which may provide further insight into the unique signatures of each specific cell type of EVs [44,45,46,47]. We have not fully evaluated the value of some of the other protein markers in this study in greater detail, including HCK- and HCF-EVs. The data at hand show that HCK-EVs do not express high levels of RAB5A, RAB11A, or CHMP2A, which suggests that HCK-EVs may not be driving endocytosis compared to other EV types [48,49]. The data at hand also show that HCF-EVs might be involved in the extrinsic prothrombin activation pathway. We also understand that additional research is required to delve into internal EV protein markers to distinguish between different EV types, such as exosomes, microvesicles, apoptotic bodies, and others. This highlights some of the complexities in understanding the protein signatures that are unique to each specific cell type, and future studies will ascertain in more detail the roles these proteins may have.

The current EV study provides evidence that HCE-, HCK-, HCF-, and HCM-EVs contain distinct cargo proteins (Table 1) that are unique to each EV type and cell of origin. The proteins identified here could provide novel markers that are unique and enriched for specific cell-derived EVs, such as WFDC2 and GPRC5A for HCE-EVs as one example. Understanding the molecular components and mechanism(s) of action will be important for future studies. We have shown evidence that the EV cargo is distinct, which could provide a basis for beneficial findings in other cornea-related EV studies.

## 4. Materials and Methods

### 4.1. Cell Culture

The immortalized Araki-Sasaki corneal epithelial cell line HCE-TJ [19] was cultured and maintained in keratinocyte SFM (Gibco, Grand Island, NY, USA), supplemented with 5 ng/mL epithelial growth factor (Gibco), 0.05 mg/mL bovine pituitary extract (Gibco), and 1x antibiotic–antimycotic (Gibco). The cells were grown at 37 °C in a 5% CO_2_ environment.

Human corneas were obtained from the National Disease Research Interchange (NDRI; Philadelphia, PA, USA). All research adhered to the tenets of the Declaration of Helsinki. Cells were cultured as previously described After isolation, cells were plated in 6-well plates and grown to 75% confluency in Eagle’s minimum essential medium (EMEM) (American Type Culture Collection; Manassas, VA, USA) with either 1% fetal bovine serum (FBS; Atlanta Biologicals; Flowery Branch, GA, USA) for HCK media or 10% FBS for HCF media, along with 1x antibiotic–antimycotic (Gibco). In this study, HCKs refer to cells maintained in 1% FBS, indicating the preservation of a keratocyte-like phenotype [50,51,52]. HCFs are cells exposed to 10% FBS, reflecting a distinct phenotype from normal keratocytes, as previously shown [53]. HCMs are derived from HCFs treated with 2 ng/mL of TGF-β1, resulting in HCMs with αSMA+ fibers [5,14,54,55].

### 4.2. Extracellular Vesicle (EV) Isolation

Cell-conditioned media (CM) from HCE, HCK, HCF, and HCM cultures were collected after a 36 h incubation in serum-free media to isolate EVs. The CM underwent differential centrifugation at 300× *g* for 10 min, then at 3000× *g* for 10 min, and at 13,000× *g* for 30 min. CM were concentrated using a Centricon^®^ Plus-70 centrifugal filter unit with a 100 kDa MW cutoff (MilliporeSigma, Burlington, MA, USA). The concentrated CM underwent ultracentrifugation at 110,000× *g* for 1 h and 10 min at 4 °C using a Beckman type 50.2 Ti rotor (Beckman Coulter, Brea, CA, USA) in an Optima LE-80K ultracentrifuge. The pellet was resuspended in phosphate-buffered saline (PBS; Gibco) and centrifuged again at 110,000× *g* for 1 h and 10 min at 4 °C. The final pellet was stored at −80 °C until further use.

### 4.3. Western Blot

Protein isolation and Western blot analyses were performed as previously described [2,56]. Proteins were extracted from EVs using RIPA buffer (10 mM Tris, 150 nM NaCl, 1% deoxycholic acid, 1% Triton X, 0.1% SDS, and 1 mM EDTA) plus protease inhibitors (aprotinin, PMSF, and sodium orthovanadate) and in non-reducing conditions. Protein concentration was determined using a Pierce™ bicinchoninic acid (BCA) protein assay kit (ThermoFisher Scientific, Waltham, MA, USA). Protein samples (20 μg/lane) were loaded on a 4–20% polyacrylamide gel (Bio-Rad, Hercules, CA, USA). Membranes were blocked in PBS 0.05% Tween20^®^ 5% milk for 2 h, then probed overnight with primary antibodies: CD81 (Sc-7637; Santa Cruz, CA, USA), integrin αV (ITGAV) (Sc-9969; Santa Cruz), fibronectin (FN1) (Sc-8422; Santa Cruz), and GM130 (#12480; Cell Signaling, Danvers, MA, USA). The secondary antibodies (1:2000, donkey anti-mouse IRDye 800CW: LI-COR Biosciences, Lincoln, NE, USA; donkey anti-rabbit IRDye 680RD, LI-COR Biosciences) were incubated with the membrane for 1 h at room temperature (RT). All antibodies were diluted as recommended by manufacturers. Membranes were imaged using a fluorescence scanner (Odyssey v.3.0, LI-COR Biosciences).

### 4.4. Nanoparticle Tracking Analysis (NTA) and Zeta Potential Measurements

All EV samples were diluted in PBS to a final volume of 1 mL. Optimal measurement concentrations were determined by pretesting to achieve an ideal particle count per frame (140–200 particles/frame). The manufacturer’s default software settings for EVs or nanospheres were then selected accordingly. For each measurement, three cycles were performed by scanning 11 cell positions and capturing 30 frames per position with the following settings: focus: autofocus; camera sensitivity: 75 for all samples; shutter: 100; scattering intensity: automatically detected; cell temperature: 25 °C. After capture, the videos were analyzed using ZetaView 8.04.02 SP2 with specific analysis parameters: maximum area: 1000, minimum area: 5, minimum brightness: 25. Hardware included an embedded 40 mW laser at 488 nm and a CMOS camera. The number of completed tracks in NTA measurements consistently exceeded the recommended minimum of 1000 to reduce data skewing by single large particles [57]. The zeta (ζ) potential of EVs was measured using the same settings as described for NTA, and the ZetaView software 8.04.02 was employed for data collection and analysis.

### 4.5. Transmission Electron Microscopy (TEM)

EVs were fixed and imaged by transmission electron microscopy (TEM) to assess EV morphology as described in [5,58]. Paraformaldehyde (4% *w*/*v*) in PBS (Gibco) was resuspended with the EV pellet for 30 min at RT for fixation. A 5 μL solution of the fixed EV pellet was added to a formvar/carbon-coated grid (Electron Microscopy Sciences, Hatfield, PA, USA), followed by a 20 min incubation to allow EVs to adhere to the grid surface. The grids were washed in drops of PBS to remove residual PFA, followed by resuspension in 1% *v*/*v* glutaraldehyde in PBS for 5 min. Residual glutaraldehyde was removed by resuspending the grid in water. The grids were transferred to a uranyl oxalate solution followed by a 10 min incubation with a methyl cellulose solution for contrast. Adsorbed EVs on the grids were dried out prior to examination by TEM imaging (JEM-1220 TEM: JEOL USA, Peabody, MA, USA).

### 4.6. Tandem Mass Tag (TMT) Mass Spectrometry

HCE-, HCK-, HCF- and HCM-EVs were lysed with lysis buffer and protein concentrations were measured. All samples were subjected to enzymatic digestion. All 4 samples (in duplicate) were labeled with a Tandem Mass Tag™ (TMT™) 10-plex for quantitative proteomic experiments. After the labeling step, a small fraction of each sample was pooled together to examine the labeling efficiency.

#### 4.6.1. Sample Protein Digestion and Labeling

RapiGest (Waters, Milford, MA, USA) was resuspended and added to each EV sample to achieve a final concentration of 1% (mass/volume). To complete lysis, the samples were sonicated for 10 s at 20% amplitude using a Q500 Sonicator (QSonica, Newtown, CT, USA) and then heated at 90 °C for 5 min. The samples were centrifuged using a tabletop centrifuge, and protein concentrations were measured with a BCA assay (ThermoFisher Scientific). A total of 15 μg of EV protein from each sample was normalized to a final volume of 400 μL using 100 mM HEPES buffer (pH 8.5). To each sample, 40 μL of 10 mM dithiothreitol (DTT) was added, followed by incubation at 60 °C for 15 min in an Eppendorf Thermomixer^®^ C (Eppendorf, Hamburg, Germany). Next, 40 μL of 20 mM iodoacetamide (IAM) was added at room temperature and incubated in the dark for 20 min. To quench any excess IAM, 10 s mM DTT was added. Each sample then received 450 μL of water and 30 μL of 100 mM HEPES buffer (pH 8.5), reducing the RapiGest concentration to below 0.5% for enzymatic digestion. Trypsin–LysC (15 μL at 0.1 μg/μL, ThermoFisher Scientific) was added to each sample, which was then shaken at 37 °C in the Eppendorf Thermomixer^®^ C. An additional 5 μL of trypsin–LysC was added after 12 h of incubation. After 16 h, the trypsin reaction was quenched with 100 μL of 10% trifluoroacetic acid (TFA). Each sample was cleaned using C18 stage tips (PhyNexus Inc., San Jose, CA, USA), and the eluates were dried in a Speedvac SPD120 concentrator (ThermoFisher Scientific) in preparation for TMT labeling.

#### 4.6.2. Sample Labeling and High pH Fractionation

Each dried sample was resuspended in 40 μL of 100 mM HEPES buffer (pH 8.5), gently vortexed, and centrifuged. TMT labels were dissolved in 80 μL of acetonitrile (ACN; Optima™ LC/MS Grade; ThermoFisher Scientific), and 20 μL of this TMT reagent was added to each EV sample. The samples were then incubated at room temperature for 1 h. For quality control, 1 μL of each TMT-labeled sample was mixed and analyzed to check the labeling efficiency. The label check mixture was combined with 80 μL of reconstitution buffer (1% formic acid) and analyzed by liquid chromatography–mass spectrometry (LC/MS), confirming a labeling efficiency of approximately 99%. To terminate the TMT reaction, 15 μL of 5% hydroxylamine solution was added to each TMT-labeled sample. The samples were then pooled and dried using the Thermo Speedvac SPD120 (ThermoFisher Scientific).

The dried TMT-labeled mixture was reconstituted with 80 μL of deionized water and fractionated using high-pH reverse-phase high-performance liquid chromatography (HPLC; ThermoFisher Scientific). Over a 75 min period, 96 fractions were collected. Twelve selected fractions were combined into one, resulting in a total of eight final fractions. These combined fractions were then dried and desalted using C18 stage tips (PhyNexus Inc.).

All fractionated samples were analyzed using the Ultimate™ 3000 nanoflow HPLC system (ThermoFisher Scientific), followed by Orbitrap Eclipse Tribrid mass spectrometry (ThermoFisher Scientific). A Nanospray Flex™ ion source (ThermoFisher Scientific) was paired with a PRSO-V2 column oven (Sonation, Biberach, Germany) to heat a PicoFrit^®^ nanocolumn (100 μm × 250 mm × 15 μm tip; New Objective, Littleton, MA, USA) for peptide separation. The nanoLC method was water–CAN-based, with a runtime of 150 min and a flow rate of 0.35 μL/min. For each TMT fraction, all TMT-labeled peptides were first captured on a trap column (ThermoFisher Scientific) before being transferred to the separation nanocolumn via the mobile phase. A TMT-specific MS2-based method on the Orbitrap Eclipse was employed to sequence the TMT peptides as they eluted from the nanocolumn. For the full MS, a resolution of 120,000 was used with an AGC target of 3E6, and the scan range was 300 m/z to 1500 m/z. For the dd-MS2 (MS/MS), a resolution of 60,000 was applied with an AGC target of 1E5. The isolation window was set to 0.7 Da, with a fixed first mass of 110.0 Da. The normalized collision energy (NCE) was set to 32 with a 15-cycle loop.

#### 4.6.3. Mass Spectrometry Analysis

The collected LC-MS data were analyzed using Proteome Discoverer 2.4 (ThermoFisher Scientific). Since all peptides were labeled with TMT tags, TMT quantitative proteomic searches were conducted. These searches were performed using the SEQUEST HT node, with a mass tolerance of 20 ppm for MS1 and 0.05 Da for MS2. The Homo sapiens database (UP000005640) from Swiss-Prot was utilized. The Percolator node was applied for peptide false-discovery rate (FDR) filtering, with strict settings at 0.01 and relaxed settings at 0.05. The abundance of TMT-labeled peptides was normalized by the total peptide abundance.

### 4.7. Qiagen™ Ingenuity Pathway Analysis (IPA)

Qiagen™ IPA software Version 2022.4 (Qiagen, Germantown, MD, USA) explored proteins enriched in distinct disease function pathways. Proteins that exhibited a false-discovery rate adjusted *p*-value < 0.05 and log2 fold change >0.5 or <−0.5 among one another—HCE-EVs, HCK-EVs, HCF-EVs, and HCM-EVs—were uploaded to IPA for primary analysis. Pairwise comparisons for each group were then combined into a final analysis using the IPA comparison module. Only pathways showing a Z score >2 or <−2 among the final combined pairwise comparisons were included, in which positive and negative Z scores represented activated and inhibited pathways, respectively. Protein lists for relevant pathways were then downloaded from IPA for further analysis and visualization into heatmap configuration.

### 4.8. Statistics

Data are reported as means + SEM unless stated otherwise. Differences between experimental groups was compared by ANOVA followed by Bonferroni’s multiple comparison test using GraphPad Prism (Version 8.4.2; GraphPad, San Diego, CA, USA).

## 5. Conclusions

The current proteomic corneal EV study provides evidence that HCE-, HCF-, HCK- and HCM-EVs express a unique array of distinct proteins that could be involved in pathways identified by IPA functional analysis. The proteins identified here could serve as unique protein markers for each specific corneal EV subtype and could be harnessed towards biomarker discovery by characterizing proteins of interest. Understanding the molecular components and their prospective mechanism(s) of action will be vital for future studies, but we demonstrate that the corneal EV is distinct and could provide beneficial findings for cornea-related functional and EV biomarker studies.

## Figures and Tables

**Table 1:** Proposed HCE-, HCF-, HCK-, and HCM-EV enriched protein markers and functional IPA.

EV Type	Enriched Protein Markers	IPA
HCE-EV	GPRC5A, KRT7, KRT8, KRT18, KRT19, WFDC2, FLOT2	EIF Signaling Pathway
HCK-EV	APOA1, APOM, DMKN, IAH1, AGBL5, KRT13	Acute-Phase Response Signaling
HCF-EV	FDXR, APEX1, LAMP1, PARP1, VAMP8	Extrinsic Prothrombic Activation, Coagulation Pathway
HCM-EV	COL6A1, COL6A2, COL6A3, MMP1, MMP2, TIMP1, TIMP2 CXCL6, CXCL12	Fibrosis Pathway

## Data Availability

The data are now available on the open science framework (OSF) to view: https://doi.org/10.17605/OSF.IO/8MCSX.

## Supplementary Materials

The following supporting information can be downloaded at https://www.mdpi.com/article/10.3390/ijms251910338/s1.
