# Supplementary figures and images for: Proteomic Characterization of Corneal Epithelial and Stromal Cell-Derived Extracellular Vesicles
Source: Int J Mol Sci. 2024 Sep 26;25(19):10338. doi: 10.3390/ijms251910338 (PMC11477500; doi:10.3390/ijms251910338)

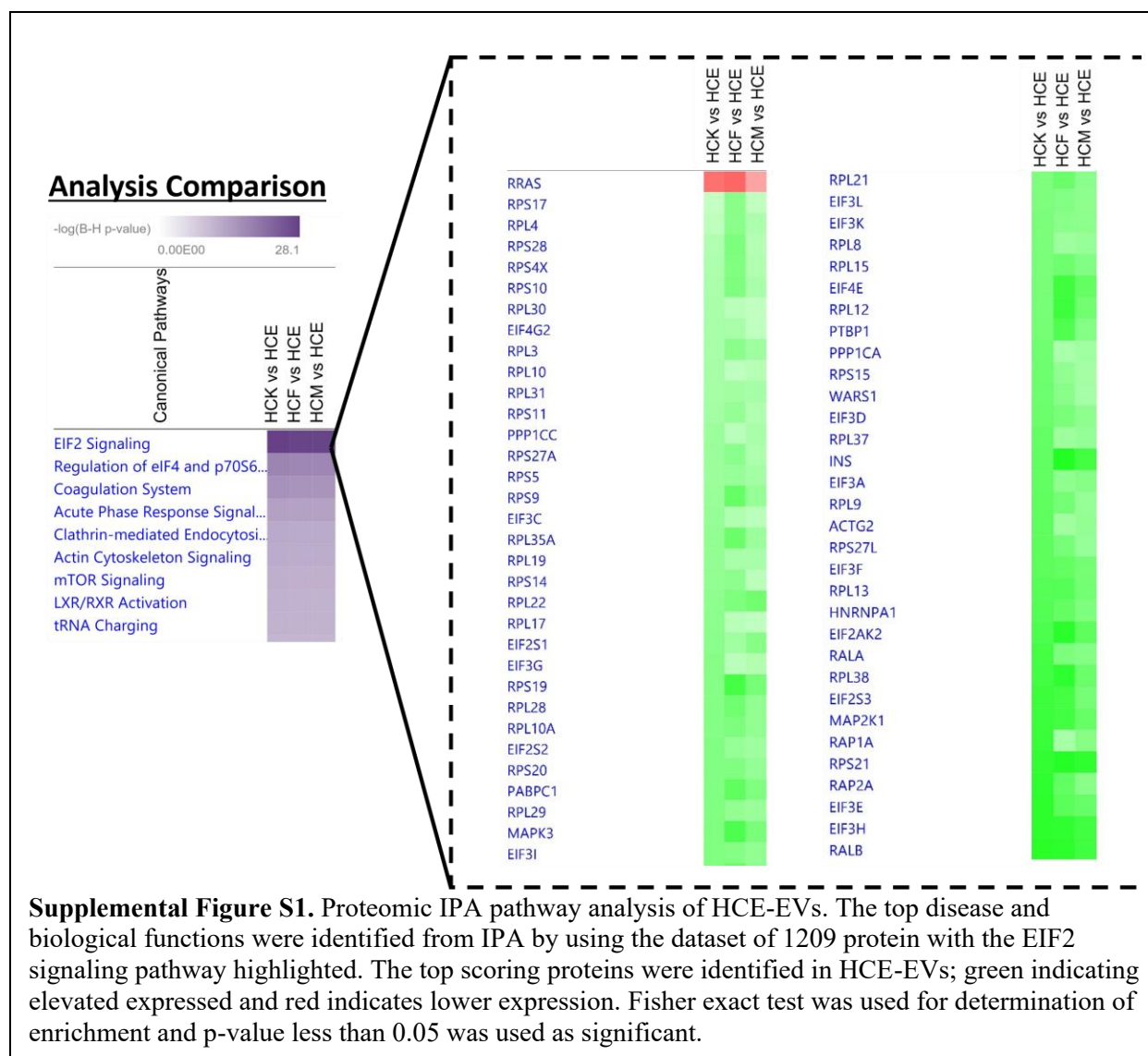

Supplement: Supplementary file 1 [file ijms-25-10338-s001.zip › Figure S1.pdf]
